# Infection with New York Orthohantavirus and Associated Respiratory Failure and Multiple Cerebral Complications

**DOI:** 10.3201/eid2506.181966

**Published:** 2019-06

**Authors:** Rajeev Fernando, David Capone, Susan Elrich, Raymond Mantovani, Luther Quarles, Alison D’Amato, Nathan Lowe, Ashwin Malhotra, Teresa Khoo, Sara Zufan, Maria Morales-Betoulle, Shelley M. Brown, Deborah Cannon, James C. Graziano, John D. Klena, Shannon Whitmer, Stuart T. Nichol, Paul Strachan, Bernard C. Camins, Luis A. Marcos

**Affiliations:** Stony Brook Southampton Hospital, Southampton, New York, USA (R. Fernando, D. Capone, S. Elrich, R. Mantovani, L. Quarles III, A. D’Amato, N. Lowe);; New York Presbyterian Hospital/Weill Cornell Medical Center, New York, New York, USA (A. Malhotra);; Stony Brook University, Stony Brook, New York, USA (A. Malhotra, T. Khoo, P. Strachan, L.A. Marcos);; Centers for Disease Control and Prevention, Atlanta, Georgia, USA (S. Zufan, M. Morales-Betoulle, S.M. Brown, D. Cannon, J.C. Graziano, J.D. Klena, S. Whitmer, S.T. Nichol);; Icahn School of Medicine at Mount Sinai, New York (B.C. Camins)

**Keywords:** New York orthohantavirus, viruses, hantavirus, hantavirus pulmonary syndrome, respiratory failure, multiple cerebral complications, vector-borne infections, zoonoses, Long Island, New York, United States

## Abstract

We report a case of infection with New York orthohantavirus in a woman who showed renal impairment and hemorrhage, complicated by hydrocephalus, in Long Island, New York, USA. Phylogenetic analysis showed that this virus was genetically similar to a New York orthohantavirus isolated in the same region during 1993.

In the United States, New York orthohantavirus (NYV) is carried by the white-footed mouse (*Peromyscus leucopus*). During 1993–1995, human cases of NYV infection were reported in persons who visited or resided on Long Island, New York, USA (*1*). Since that time, no other cases have been reported on Long Island. We report an NYV infection with unusual clinical manifestations.

A 52-year-old woman from the east end of Long Island came to an emergency department in May 2017 because of fevers, headaches, myalgias, vomiting, somnolence, and lethargy for 3 days. She had a history of tick exposure and had recently cleaned her basement, where she reported observing mice droppings.

The patient had a core temperature of 102°F, a heart rate of 108 beats/min, a respiration rate of 20 breaths/min, and a blood pressure of 94/56 mm Hg. Her oxygen saturation was 95% on room air. Laboratory tests showed the following results: leukocytes 4,500 cells/mm^3^, platelets 102,000 platelets/mm^3^, aspartate aminotransferase 355 U/L, alanine aminotransferase 180 U/L, procalcitonin 4.3 ng/mL, erythrocyte sedimentation rate 2 mm/h, and C-reactive protein 4.7 mg/dL. A chest radiograph showed clear lung fields. A lumbar puncture showed an opening pressure of 45 cm of H_2_O (reference range 8–15 cm H_2_O), 1 nucleated cell (reference value <5/high-powered field), glucose 152 mg/dL, and protein 31 mg/dL.

On day 4 postonset, her condition rapidly deteriorated. A second chest radiograph showed developing pulmonary congestion, and she required intubation and mechanical ventilation. She also had severe thrombocytopenia (18,000/platelets mm^3^), acute kidney injury, proteinuria and microhematuria (peak creatinine 2.3 mg/dL, urinary protein 30 mg/dL, 50 erythrocytes/high-powered field), worsening transaminitis (aspartate aminotransferase 329 U/L, alanine aminotransferase 258 U/L), and an increased lactate dehydrogenase (769 U/L). A computed tomography (CT) scan of the lungs showed bilateral pleural effusions (day 6 postonset). Because a CT scan of the head showed cerebral edema, the patient was transferred to a tertiary care hospital (day 7 postonset).

Serologic test results were negative for Powassan virus, *Ehrlichia* spp., *Anaplasma* spp., Lyme disease, and Rocky Mountain spotted fever. Results of a hantavirus IgM and IgG ELISA (recognizing the nucleocapsid protein common to all hantaviruses) were positive (Associated Regional and University Pathologists Laboratories, https://www.aruplab.com). An acute hantavirus infection was confirmed by the Centers for Disease Control and Prevention (Atlanta, GA, USA) by ELISA and identified antibodies that cross-reacted with Sin Nombre virus antigens (serum titers: IgM 1:6,400 and IgG 1:400). Orthohantavirus RNA was also detected by using reverse transcription PCR specific for the large RNA segment (*2*).

Although the patient’s condition improved on day 14 postonset, she had 3 episodes of witnessed tonic–clonic seizures. A CT scan of the head showed an acute intraparenchymal hemorrhage (platelet count range 37,000/μL–57,000/μL) in the left basal ganglia with intraventricular extension into the left lateral and third ventricles and hydrocephalus that required placement of an emergent external ventricular drain. Blood and cerebrospinal fluid (CSF) collected at day 15 postonset had undetectable levels of virus RNA but IgM (serum 1:6,400, CSF 1:20) and IgG (serum 1:6,400, CSF 1:20) antibody titers had increased. She was discharged on day 26 postonset and given anticonvulsant therapy. One year later, the patient was asymptomatic.

We sequenced hantavirus RNA isolated from the initial blood specimen by using Sanger and unbiased next-generation sequencing (Figure). Phylogenetic analysis of a partial genome inferred that the virus had a most recent common ancestor with an NYV collected in 1994 from a white-footed mouse from Shelter Island, NY (*3*). Given the inferred evolutionary relationship, our data are consistent with disease transmission likely occurring after contact with excreta from a local rodent. It is unclear if NYV (formerly classified as a distinct species) might have caused the neurologic complications (hydrocephalus and intracranial bleeding) for this case. Neurologic signs and symptoms did not develop for previous cases of infection with NYV (*1*).

Hantavirus pulmonary syndrome is a severe respiratory illness caused by infection with New World hantaviruses (*4*). During 1993–2015, a total of 688 cases of hantavirus pulmonary syndrome were reported in the United States (case-fatality rate 36%) (*5*). We report an orthohantavirus infection that progressed to respiratory failure and renal impairment on day 4 postonset, which preceded cerebral complications on day 14 postonset. Brain complications, including subarachnoid (*6*), pituitary hemorrhage (*7*), and encephalitis (*8*), with orthohantavirus infections have been reported. The direct effect of the virus into the brain has been demonstrated in an animal model (*9*), which raises the question whether intracranial bleeding for this case-patient was associated with endothelial damage directly from the virus infection. 

In conclusion, we report an orthohantavirus infection in New York that caused intracranial bleeding and hydrocephalus that required an emergent surgical intervention. Because orthohantaviruses are endemic to North America and several strains/species have not been fully characterized, it is essential that clinicians recognize and be aware of other clinical manifestations of these infections (e.g., kidney injury), which are often indicators of subsequent complications.

**Figure:**
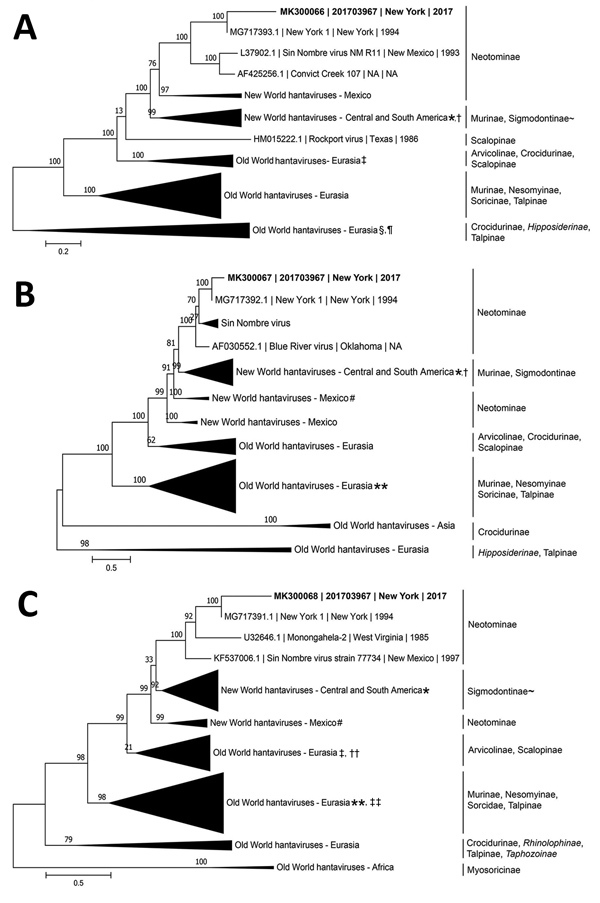
Inference of phylogenetic relationships for A) large, B) medium, and C) small RNA segments of orthohantaviruses by using representative full-genome sequences in study of infection with New York orthohantavirus and associated respiratory failure and multiple cerebral complications. Partial sequences from the case in New York during 2017 (specimen no. 201703967), are indicated in bold (GenBank accession no. MK300066–8). Genome coverage for the 201703967 small, medium, and large RNA segments are 12%, 30%, and 5%, respectively. Evolutionary history was inferred by using a maximum-likelihood method based on the subtree pruning and regrafting model with the general time-reversible + gamma (n = 4) nucleotide substitution model, and branch support was calculated by using the approximate likelihood ratio test method. Branch lengths are measured in number of nucleotide substitutions per site (scale bars). Clades are collapsed by region for clarity, and clades of interest are annotated with reservoir subfamily. Clades with incomplete reservoir information are annotated with ~, and bat subfamilies are shown in italics. Sequences collected outside a specified geographic region are denoted by a symbol. *Black Creek Canal virus, USA; †Bayou virus, USA; ‡Prospect Hill virus, USA; §Kilimanjaro virus, Tanzania; ¶Uluguru virus, Tanzania; #Limestone Canyon virus, USA; **Sangoussa virus, Guinea; ††Rockport virus, USA; ‡‡Jemez Springs virus, USA.
